# Sensitivity analyses for trials with missing data, assuming missing not at random mechanisms

**DOI:** 10.1186/1745-6215-14-S1-O97

**Published:** 2013-11-29

**Authors:** Baptiste Leurent, Mike Crawford, Hazel Gilbert, Richard Morris, Mike Sweeting, Irwin Nazareth

**Affiliations:** 1 UCL PRIMENT CTU, London, UK; 2 UCL Department of Primary Care and Population Health, London, UK; 3 MRC Biostatistics Unit, Cambridge, UK; 4 Centre for Mental Health, Imperial College London, London, UK

## 

In randomised trials with missing data, it is not uncommon for the observation of the outcome to depend on the outcome itself. For example in behavioural trials on smoking cessation, weight loss, or alcohol reduction, unsuccessful participants may be less willing to disclose their outcome than those that are more successful. These Missing Not At Random (MNAR) data are problematic because they can bias the estimate of the treatment effect, and because the observed data do not provide any information on the likelihood of such a mechanism. Trialists in each field tend to favour one approach regarding missing data. For example in smoking cessation trials non-responders are usually assumed to be still smoking, or in weight loss trials, baseline or last observation carried forward are typically used. The assumptions made for each of these analyses, and the extent to which departure from these assumptions could affect the trial results, are often ignored. We propose a simple technique to perform sensitivity analyses of randomised trials where MNAR outcomes are expected. It is based on a *a-priori* discussion with investigators regarding the plausible missing data mechanisms, and an evaluation of the treatment effect under scenarios covering a range of assumptions, including possible difference in mechanism between arms. Results under each assumption are tabulated alongside indicators of the plausibility of each assumption. We will discuss the application of this approach as applied to two recent large trials, one in smoking cessation, and one in alcohol use. In both instances, this approach offered a more robust interpretation of the trial finding.

